# Distinct Neural Processes for Memorizing Form and Meaning Within Sentences

**DOI:** 10.3389/fnhum.2019.00412

**Published:** 2019-12-05

**Authors:** Matteo Mascelloni, Roberto Zamparelli, Francesco Vespignani, Thomas Gruber, Jutta L. Mueller

**Affiliations:** ^1^Institute of Cognitive Science, University of Osnabrück, Osnabrück, Germany; ^2^School of Psychology and Counselling, Faculty of Health, Queensland University of Technology, Brisbane, QLD, Australia; ^3^Institute of Health and Biomedical Innovation, Queensland University of Technology, Brisbane, QLD, Australia; ^4^Center for Mind/Brain Sciences, University of Trento, Rovereto, Italy; ^5^DIPSCO, University of Trento, Trento, Italy; ^6^Institute of Psychology, University of Osnabrück, Osnabrück, Germany

**Keywords:** sentence repetition, language production, working memory, syntax, semantics, ERP, slow wave, mental rehearsal

## Abstract

In order to memorize sentences we use both processes of language comprehension during encoding and processes of language production during maintenance. While the former processes are easily testable via controlled presentation of the input, the latter are more difficult to assess directly as language production is typically initiated and controlled internally. In the present event-related potential (ERP) study we track subvocal rehearsal of sentences, with the goal of studying the concomitant planning processes with the help of a silent cued-production task. Native German participants read different types of sentences word-by-word, then were prompted by a visual cue to silently repeat each individual word, in a *rehearsal phase*. In order to assess both local and global effects of sentence planning, we presented correct sentences, syntactically or semantically violated sentences, or random word order sequences. Semantic violations during reading elicited an N400 effect at the noun violating the selectional restrictions of the preceding verb. Syntactic violations, induced by a gender incongruency between determiner and noun, led to a P600 effect at the same position. Different ERP patterns occurred during the silent production phase. Here, semantically violated sentences elicited an early fronto-central negativity at the verb, while syntactically violated sentences elicited a late right-frontal positivity at the determiner. Random word order was accompanied by long-lasting slow waves during the production phase. The findings are consistent with models of hierarchical sentence planning and further indicate that the ongoing working memory processes are qualitatively distinct from comprehension mechanisms and neurophysiologically specific for syntactic and lexical-semantic level planning. In conclusion, active working memory maintenance of sentences is likely to comprise specific stages of sentence production that are indicated by ERP correlates of syntactic and semantic planning at the phrasal and clausal level respectively.

## Introduction

Having language at our disposal serves multiple purposes. While it undisputedly works as a means of communication between individuals, it also serves as a code for cognition within the individual (Pinker and Bloom, 1990; Bolhuis et al., 2014; Asoulin, 2016). A cognitive function which uses language in complex ways is working memory, which is conceived as the cognitive function supporting “the few temporarily active thoughts” (Cowan, 2010, p. 51). In many cases, our thoughts are verbal in nature, which is why models of working memory include some kind of language-based processes. An important mechanism included in many current models of verbal working memory is subvocal rehearsal as a mechanism of maintaining arbitrary verbal material (Baddeley and Hitch, 1974; Cowan, 1999; Camos et al., 2009). In addition to phonological information, as instantiated in subvocal rehearsal, higher order linguistic representations such as semantic and syntactic information have also been shown to contribute to working memory processing. A prime example involving both phonological/articulatory and higher order linguistic processes is working memory for sentences. Typically, the process of memorizing sentences is easy, even when verbatim recall is required (e.g., dictation). The present study aims to contribute to the understanding of how well-formed sentences are retained more efficiently and more accurately than unstructured lists of words.

It has long been known that our memory for words embedded in sentences far exceeds the typical short-term memory span of ±7 items (Brener, 1940; Miller and Selfridge, 1950; Marks and Miller, 1964). Readers experience difficulties when trying to correctly repeat a random word sequence such as “out prince swamp the of white the of are carriage horses pulling dangerous the” after reading it. The same words can easily be repeated, however, when they are organized within a sentence: “white horses are pulling the carriage of the prince out of the dangerous swamp.” This so-called “sentence superiority effect” is a very robust observation and measurable even in two-word lists (Perham et al., 2009) and meaningless “jabberwocky” sentences (Marks and Miller, 1964; Bonhage et al., 2014). It can be measured with different tasks, including recall (Baddeley et al., 2009; Allen et al., 2018) and recognition (Bonhage et al., 2014; Allen et al., 2018). Many studies have demonstrated that the sentence-superiority effect is due to rapid access to stored linguistic knowledge and conceptual/semantic processes which improve the way our memory encodes, maintains and retrieves meaningful sentences (Potter and Lombardi, 1990; Lombardi and Potter, 1992; Jefferies et al., 2004; Baddeley et al., 2009; Perham et al., 2009; Schweppe et al., 2011; Bonhage et al., 2014). Yet, how exactly subvocal rehearsal benefits from, or interacts with higher order linguistic information has not been investigated at a fine-grained level. On the one hand, there is evidence that subvocal rehearsal is dispensable if higher order linguistic information is available (e.g., syntactic and/or semantic relations), as suggested by the robustness of the sentence superiority effect in the face of articulatory suppression (Baddeley et al., 2009; Bonhage et al., 2014). On the other hand, studies comparing memory for different types of sentences provide evidence that subvocal rehearsal plays a role even for remembering well-formed sentences (Meltzer et al., 2016, 2017).

The present study tests how higher order linguistic information and subvocal rehearsal interact. This is done by investigating specifically how syntactic and semantic information contribute to sentence memory and, in particular, to subvocal rehearsal as a working memory maintenance mechanism. As this research question involves processes discussed in the working memory as well as in the language production literature, we will review studies from both fields with a specific focus on neurophysiological processes at the sentential level, i.e., working memory for sentences and sentence production.

Subvocal rehearsal is a well-investigated, yet not uncontroversial mechanism for the short-term memorization of verbal and verbalizable material, and is part of multi-component as well as process models of working memory (Cowan, 1999; Baddeley, 2003; but see Lewandowsky and Oberauer, 2015). There is ample evidence for the psychological reality of subvocal articulation processes during memory processing, even though the efficiency of such processes has been questioned (Souza and Oberauer, 2018). Early conceptualizations of short-term memory already included subvocal rehearsal as a mechanism of maintaining verbal information (Waugh and Norman, 1965; Sperling, 1967; Atkinson and Shiffrin, 1968). Evidence for such a mechanism stems, for example, from the observation that concurrent articulation of irrelevant speech (e.g., the articulation of “ne na da na ne na….”) interferes with the maintenance of verbal material (Murray, 1967). Sequences consisting of longer words are more difficult to remember than sequences consisting of shorter words (Baddeley et al., 1975, 1984). This can be explained by the additional time needed to pronounce longer words, an explanation which is supported by the observation that articulatory suppression eliminates this effect (Baddeley et al., 1984). Further, participants in memory tasks frequently report using subvocal articulation strategically (Dunlosky and Kane, 2007; Morrison et al., 2016). Instructions to rehearse word lists aloud seem to improve performance especially in participants with low working memory spans (Turley-Ames, 2003). Thus, while the role and importance of subvocal rehearsal remain debated, it clearly plays a role in short-term maintenance of arbitrary verbal information. Lastly, brain areas that are involved during overt language production, such as premotor cortex (BA6) and parts of the inferior frontal cortex (BA44) (Indefrey and Levelt, 2004; Saur et al., 2008) also play a role during the maintenance phase in verbal working memory (Chein et al., 2003; Buchsbaum and D’Esposito, 2008; Bonhage et al., 2014). For this reason, current psychological and neurocognitive approaches to working memory posit the involvement of the same cognitive and sensorimotor processes related to language production in verbal working memory tasks (Buchsbaum and D’Esposito, 2008, 2019; Acheson and MacDonald, 2009; Majerus, 2013).

While it can be reasonably assumed that processes of language production are involved in the memory retention of arbitrary verbal information, they become less important when higher-order linguistic information, such as syntactic and semantic structures within the sentence, come into play. Early studies have already proposed that memory advantages for sentences may be due to the processing of syntactic and semantic dependencies between items (Miller and Selfridge, 1950; Marks and Miller, 1964). Later studies showed that successful sentence maintenance involves neither extensive rehearsal nor attention-demanding processes, but rather relies on long-term memory representations and automatic language processing mechanisms (Jefferies et al., 2004; Baddeley et al., 2009). Accordingly, an explicit model of immediate sentence recall, the conceptual regeneration hypothesis, assumes that conceptual-semantic, but not phonological/articulatory processes are mainly involved in immediate sentence memory (Potter and Lombardi, 1990; Lombardi and Potter, 1992). The original hypothesis was based on the observation of a specific type of error during sentence recall: Synonyms of words in sentences that were presented next to the sentences were often reproduced in replacement of the correct word in the sentence (Potter and Lombardi, 1990). Later studies, however, provided a more multifaceted picture by showing in similar experimental designs that phonological and syntactic information can also interfere with sentence memory (Rummer and Schweppe, 2005; Schweppe and Rummer, 2007; Schweppe et al., 2011). Thus, it seems highly likely that linguistic codes at all levels, from articulatory to conceptual, play a certain role in immediate sentence memory.

In agreement with the findings from behavioral studies, a recent fMRI study demonstrated that working memory for sentences, compared to unstructured word sequences, involves a widely distributed network of brain areas related to semantic processing during encoding, and decreased activation of subvocal rehearsal-related areas during maintenance (Bonhage et al., 2014). This and other studies suggest that the working memory benefits during maintenance (consisting of a smaller amount of rehearsal-related activity and performance increase) may be contingent on enhanced processing costs during the encoding phase (Bor et al., 2003, 2004; Bonhage et al., 2014). In an EEG study on the memorization of sentences vs. unstructured word sequences, sentence maintenance was accompanied by reduced oscillatory power in the theta, alpha, and beta bands (Bonhage et al., 2017), frequencies which have all been related to working memory load (Jensen and Tesche, 2002), and in the case of theta oscillations, to the application of rehearsal strategies (Meltzer et al., 2017). Other electrophysiological studies have used event-related potentials (ERP) to investigate the retention of verbal material either in working memory tasks or in sentence processing tasks. Studies using working memory tasks have reported long-lasting frontal negativities for the costs of retention of verbal compared to non-verbal material (Lang et al., 1992; Ruchkin et al., 1992). While some authors have related the frontal slow waves directly to phonological rehearsal processes (Lang et al., 1992; Ruchkin et al., 1992), others, who reported similar slow waves for non-verbalizable conditions, have interpreted it as being related to attentional control of working memory contents (Bosch et al., 2001; Murphy et al., 2006). Studies assessing working memory costs during sentence processing have reported similar frontal negative shifts for sentences or sentence parts which were hypothesized to impose increased working memory processing loads (King and Kutas, 1995; Fiebach et al., 2002).

Together, neurophysiological studies on verbal working memory show that the availability of higher order linguistic information can reduce general brain activation related to subvocal rehearsal during the maintenance phase. In these studies, rehearsal is treated as a uniform function that can occur to a higher or lower degree, depending on the type of material and memory strategy. In fMRI studies, the presence of rehearsal is typically identified based on the involvement of brain regions that are usually correlated with articulation, specifically posterior inferior frontal and premotor areas (Bor et al., 2004; Bonhage et al., 2014). In EEG studies, rehearsal has been inferred from the presence of specific concurrent increased slow-wave amplitudes (Lang et al., 1992; Ruchkin et al., 1992) or certain oscillatory patterns (Griesmayr et al., 2010; Bonhage et al., 2017) in response to the processing of rehearsed material. Yet, the nature and sequence of the preparatory and execution processes during rehearsal has not been brought to light by these neurophysiological studies. A more fine-grained analysis of the processes involved in language production, if they occur, is still largely amiss. We suggest that models of sentence production are highly informative about how such processes are likely to be applied.

On-line language production has proven more difficult to investigate than language comprehension, specifically at the sentential level. This is due to the internal nature of the different stages of planning and execution in language production, which are only indirectly accessible. In general, psycholinguistic models of sentence production postulate that (i) there is a certain degree of planning ahead in sentence production and that (ii) there are separable planning stages, e.g., at the conceptual level, at the level of abstract lexical forms and at the level of concrete phonological forms (Garrett, 1975, 1982; Smedt and Kempen, 1987; Levelt, 1994). Tasks used in studies on sentence planning have to include some type of concrete instructions, often picture-based, specifying which sentence is to be produced. Many studies use different kinds of distractor items (Meyer, 1996; Wagner et al., 2010; Bürki et al., 2016; Klaus et al., 2017) or complexity manipulations (Ferreira, 1991; Smith and Wheeldon, 1999) in order to interfere with specific stages of sentence production. Longer or shorter onset latencies for production are then interpreted as reflecting either increased processing costs or facilitation during the planning stage, stemming from the corresponding manipulation.

The extent and flexibility of the planning scope, that is, how much planning ahead occurs at each stage of language production, is controversial (Martin et al., 2010; Klaus et al., 2017). The influential frame-and-slot model proposed by Garrett (1975, 1982) assumes a larger scope for abstract lexical planning compared to phonological planning, as evidenced by speech errors in the respective domains. Indeed, several studies suggest at least a phrasal scope of planning at the abstract lexical level (Smith and Wheeldon, 1999; Martin et al., 2010; Lee et al., 2013; Klaus et al., 2017). Studies testing phonological encoding during sentence planning also reported evidence for a phrasal scope of planning (Oppermann et al., 2010; Schnur, 2011), but also for a much smaller planning scope (Meyer, 1996; Wheeldon and Lahiri, 1997). One reason for such variable findings may be a certain degree of flexibility in the planning scope. Both sentence-related factors, such as sentence complexity (Ferreira, 1991; Smith and Wheeldon, 1999; Wagner et al., 2010) and non-sentence related factors, such as concurrent cognitive load (Boiteau et al., 2014; Klaus et al., 2017) seem to impact on how far in advance lexical-semantic and phonological word forms are planned. As a link between the domain of working memory and language production, sentence repetition has not only been used as a task to probe verbal working memory, but also as a way to assess the processes which occur during sentence planning. Thus, Ferreira (1991) presented participants with sentences of different syntactic complexity and showed that it took longer to initiate the production of a syntactically complex sentence compared to a less complex one. This was taken as an indication of a grammatical planning stage in which utterances are planned at a phrasal scope. In sum, studies on sentence production support incremental planning at different production levels with a tendency for a larger planning scope for higher-order linguistic levels.

A few neurophysiological studies have tackled the production of linguistic units longer than the single word. Haller et al. (2005), for example, have shown a specific contribution of Broca’s area for sentence generation from word triplets. Mere repetition of sentences has been shown to involve a network including the left hemispheric articulatory network (premotor cortex and parieto-temporal junction), semantic areas (left temporal lobe and inferior frontal cortex) as well as bilateral working-memory-related areas in the parietal and dorsolateral prefrontal cortex (cf. Majerus, 2013, for review).

There is a large number of EEG studies on language comprehension and a smaller number on language production. The initial ERP components that could be functionally related to language processing at the sentence level were the N400 component in response to semantic incongruities, discovered by Kutas and Hillyard (1980), and the P600 component in response to syntactic incongruities, discovered by Osterhout and Holcomb (1992). The N400 is a negative deflection in response to a stimulus with increased lexical or semantic processing demands and is typically related either to automatic processes at the stage of lexical access, or to later, more controlled semantic processes at the semantic integration stage (cf. Kutas and Federmeier, 2000, 2011; Lau et al., 2008, for reviews). The P600 component is a positivity typically found in response to syntactic manipulations, but also in the context of specific types of semantic violations, thus seen as an indicator of more global integration difficulties at the sentential level (Kuperberg, 2007; Friederici, 2011) or of internal monitoring of processing effort (van Herten et al., 2005; Sassenhagen et al., 2014). Electrophysiological studies on word and sentence production are fewer and have reported different effects (Pylkkänen et al., 2014; Bürki et al., 2016; Shitova et al., 2017; Blanco-Elorrieta et al., 2018). Bürki et al. (2016) reported differential ERP responses for gender congruency and phonological similarity of distractors during the production of simple determiner-noun phrases. The phonological similarity of the distractor and the noun was processed earlier than the gender congruency between distractor and target noun. This was interpreted as an indication of sequential phonological encoding, in which the encoding of the determiner follows the encoding of the noun. Pylkkänen et al. (2014) and Blanco-Elorrieta et al. (2018) conducted several MEG studies on the production of simple two-word adjective-noun phrases and found effects for semantic composition about 200 ms after a production cue. The effects, which they related to the stage of lexical access during production, could be localized to the anterolateral temporal and to the ventro-medial prefrontal cortex. The complexity of the phrases produced as well as the need to switch between different phrase types has been found to increase the amplitude of the P3 component (Shitova et al., 2017). The P3 component is a positive potential starting at around 300 ms after stimulus onset, which has been related to domain-general processes of context updating and cortical reorientation (Nieuwenhuis et al., 2005; Polich, 2007). In sum, the typical ERP components reported in tasks that involve word production at the sentence level comprise both a negativity, related to lexical-semantic processes, and a positivity (P3), reflecting more general processing costs relating to production planning.

## The Present Study

Our goal was to investigate how semantic and syntactic information is used during repetition of sentences in a working memory task. We assumed that subvocal sentence repetition includes core processes of sentence production, specifically conceptual, abstract lexical and phonological planning stages in addition to the silent articulation processes. This is in alignment with widespread views on sentence repetition assuming that many different language skills relating to comprehension and production contribute to the correct repetition of sentences (Lombardi and Potter, 1992; cf. Acheson and MacDonald, 2009; Klem et al., 2015). To test this assumption, we measured ERPs as a response to unstructured word sequences vs. sentences as well as ERPs in response to more subtle linguistic violations, i.e., local semantic and syntactic anomalies. We presented participants with variants of German declarative sentences consisting of a subject, a verb, a direct object and an adverbial expression. Importantly, the violations in the semantic and syntactic anomaly condition both occurred in the same position, namely at the direct object noun. In Table 1, example strings are listed for each condition.

**TABLE 1:** Examples of the stimulus material by condition.

**Condition**	**Sentence**
(A) Correct	*Die Frau bindet den Schuh im Flur* *The woman ties the [M] shoe in the hallway*
(B) Semantic	*Die Frau* ***steuert*** *den Schuh im Flur* *The woman* ***navigates*** *the[M] shoe in the hallway*
(C) Syntactic	*^∗^Die Frau bindet* ***das[N]*** *Schuh[M] im Flur* *^∗^The woman ties* ***the[N]*** *shoe in the hallway*
(D) Random word order	*^∗^Frau den die Flur bindet Schuh im* *^∗^woman the hallway ties shoe [in the]*

*M, masculine; N, neuter. Violation position is underlined in each sentence except D. ^∗^Ungrammatical sentence. Bold words refer “violation.”*

According to working memory-based models for sentence repetition, we assumed that the high working memory load incurred by unstructured word sequences would elicit long-lasting slow-waves reflecting increased verbal working memory loads (Ruchkin et al., 1997) or non-verbal domain-general memory maintenance strategies (Bosch et al., 2001) during subvocal rehearsal. Further, based on the conjecture outlined above that sentence repetition rests to a large degree on normal sentence production, we assumed more local and violation-type-specific processing costs for the semantically and syntactically manipulated sentences. Specifically, we expected processing costs reflecting different scopes of advance planning for lexical-semantic and syntactic information. As the selection of abstract lexical information and concrete determiner forms have been related to different planning stages (e.g., Bürki et al., 2016), we expected processing costs at an earlier position in the sentence for the semantic compared to the syntactic violation condition. Corresponding to a phrasal planning scope in the abstract lexical stage, we expected semantic processing costs time-locked to the verb onset in semantically anomalous verb phrases. For the syntactic condition, we expected difficulties at a later planning stage, the level of morphophonological encoding. This is based on the observation that determiners are planned together with or even after the corresponding nouns (cf. Bürki et al., 2016). Thus, we expected that the gender incongruency of the noun would modify the ERP time-locked to the rehearsal cue for the preceding determiner. Due to the explorative nature of the study, we did not have specific hypotheses about the polarity and distribution of the ERP components to be expected.

The initial reading phase served as a control condition to make sure that both our semantic and syntactic violations lead to specific processing difficulties at the same target point in the sentence, namely the direct object noun. At this position, we expected an N400 component for the semantic anomaly and a P600 component for the syntactic anomaly, reflecting the functional distinction between both types of processes.

## Materials and Methods

### Participants

Twenty-six native German participants (university students) volunteered for the study. The participants (15 female and 11 male) were between 18 and 28 years old (mean age = 22.5 years; *SD* = 2.51), all right-handed and native speakers of German. Each participant took part in two separate sessions. Two participants (one male and one female) had to be excluded from analysis, one due to technical issues during the measurement, and one because of a clinical diagnosis of dyslexia, which the experimenter was only informed about after the experiment. None of the remaining 24 participants reported any recent history of neurological or psychological disorders and none of them were subject to any medical treatments or under the influence of drugs or alcohol at the time of the experiment. All participants gave written informed consent in accordance with the declaration of Helsinki (World Medical Association, 2013) and received a written confirmation of participation.

In order to test for working memory performance, a Wechsler digit span test, forward and backward, was performed on all subjects. The mean forward span was 6.75 with a standard deviation of 1.25 (max. span = 9), while the mean score was 8.75 with *SD* = 1.82 (max. score = 14); for the backward version, the mean span was 5.20 with *SD* = 1.28 (max. span = 8) and the mean score was 7.25 with *SD* = 1.89 (max. score = 14). Participants with a forward span superior to 6 ± 1 are considered normal, for the backward span the typical range is 5 ± 1 (Peña-Casanova et al., 2009). All participants in the study fell in the normal range.

### Stimuli

The stimulus material consisted of German declarative sentences composed of seven words each. All stimuli followed the same syntactic structure exemplified in Table 1. The experiment comprised four different conditions (three violation conditions and one control); each condition consisted of 90 items, resulting in a total of 360 stimuli. In addition, the experiment included 18 rehearsal check items which were used to ensure the participants were engaged in rehearsal. Those sentences followed the same pattern as the experimental material with 9 correct sentences and 3 sentences for each of the violations. As a control condition, grammatical sentences (as shown in Table 1) were used as a baseline for comparison with the other conditions (for a complete list of the stimuli, please refer to the Supplementary Material).

In the semantic-mismatch condition, the verb from the control was substituted by a verb that agreed in meaning with the subject (e.g., *Die Frau bindet den Schuh*… *[The woman ties the shoe*…*])*, but not with the object of the sentence (e.g., *Die Frau steuert ^∗^den Schuh [The woman navigates ^∗^the shoe*…*]*). For the morpho-syntactic violation condition, the article of the object was modified, creating a gender disagreement between article and noun (e.g., ^∗^*das Schuh [*the_(neut.)_ shoe_(masc.)_]) as in e.g., Gunter et al. (2000). Since the female article *die* is the same as the plural article used for all grammatical genders in German, only masculine and neuter nouns were used in object position to avoid eliciting a response to number agreement mismatch rather than to the intended gender disagreement. For both the semantic and the syntactic mismatch condition, the violations occurred once the object noun of the sentence was encountered. The fourth and final condition comprised strings of words that were constructed by randomizing the word order of each individual sentence. This randomization was constrained in a way so that across the whole sentence, no more than two consecutive words appeared in a syntactically permissible sequence (i.e., the violation became apparent at the third word at the latest). In this condition, the first word presented was not capitalized unless it was a noun (since nouns are always capitalized in German). The previously mentioned 18 additional stimulus sentences for the rehearsal check were generated based on the same pattern of the four conditions outlined above (with a distribution of nine grammatical control sentences and three ungrammatical sentences per violation condition); however, each of these sentences were composed of new vocabulary, hence the ungrammatical sentences were not based on the grammatical control condition.

Each participant was presented with a total of 198 sentences (90 control sentences, 30 items per violation condition, plus the 18 rehearsal check sentences), which were divided equally into 99 stimuli per session. This distribution allowed for two versions of the same sentence to be shown to each participant, with a delay of at least 5 days between sessions, reducing the risk of potential repetition effects. The non-control stimuli were divided into three different lists using a Latin square design. Six sets of two lists (list A and B) were prepared. The lists were pseudo-randomized so that each condition would not appear more than three times in a row. Each subject was presented with one set (one list per session). List A and List B have been counterbalanced across subjects.

### Software and Hardware

Both stimulus presentation and behavioral data acquisition were performed with MATLAB (Version R2017a, Mathworks^®^, Natick, MA, United States) using the Psychophysics Toolbox extension (Brainard, 1997). A USB microphone was used to acquire the sound response while the button press data were acquired using a response pad from The Black Box ToolKit Ltd.

### EEG Recording and Electrodes

The EEG was recorded continuously using a TMSi 72 Refa amplifier and an EEG gel head cap by TMSi (TMSI B.V., Netherlands), using the 5% system with 64 channels (Oostenveld and Praamstra, 2001). EEG data were recorded with the TMSi Polybench software (TMSI B. V., Netherlands). The ground electrode was placed on the collar bone. The EOG was recorded using two bipolar electrophysiological inputs (BIP), the first one (EOGV) was positioned above and below the left eye, the second one (EOGH) was positioned close to the outer canthi of the left and the right eye. The impedance of all electrodes was kept below 5 Ω. The signals were acquired with a sample rate of 512 Hz with an online average reference.

### Experimental Procedure

The experiment was distributed across two sessions with identical experimental procedures. After the preparation process (30–40 min on average), participants were seated in a comfortable chair with a distance of 60 cm between the nasion and the screen. The instructions, as well as a block of five practice trials were presented to each participant, then they began the actual experiment.

**FIGURE 1:**
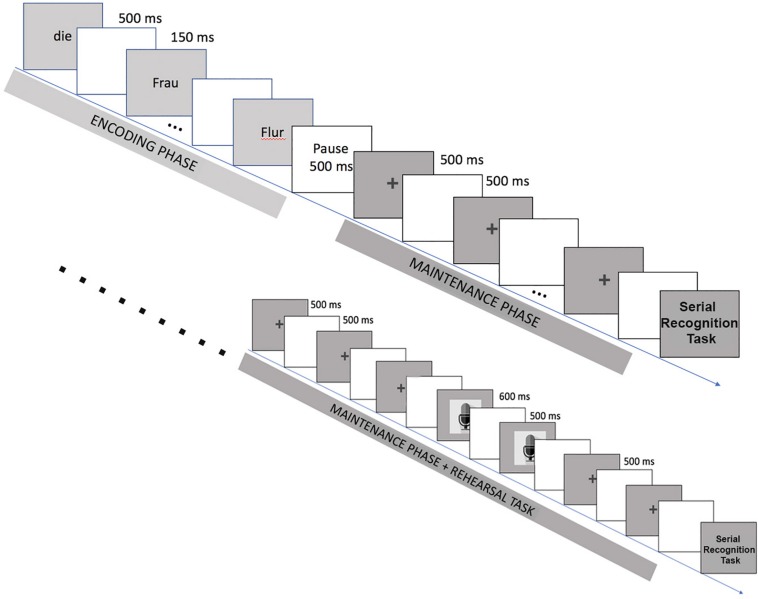
Standard Trial Sequence: Each trial sequence comprises a reading phase and a thinking phase, followed by a forced-choice decision task. The reading phase consists of a word-by-word presentation of written stimuli. During the following rehearsal phase, fixation crosses trigger silent word-by-word rehearsal of the previously presented sentence. The decision task consists of judging whether a given two-word combination appeared in the memorized sentence or not, which is indicated by pressing either of two buttons. Below: Rehearsal Check Trial Sequence: modified version of the standard trial sequence including cues for overt articulation (microphone image) of some of the memorized words.

In the standard trial sequence (Figure 1), the stimulus sentences were presented in a word-by-word manner, each word appearing on the screen for 500 ms with a blank screen being shown for 150 ms in between words. Subjects were instructed to silently read the words that appeared on the screen. After the final word of each sentence, a blank screen was presented for 500 ms. Participants were instructed to silently repeat each of the previously encountered words, precisely in the same order as they had been shown in the reading phase. After the pause, a fixation cross (+) appeared on the screen for 500 ms, followed by another blank screen (500 ms). This fixation cross/blank screen sequence was repeated seven times after each sentence, once for every previously presented word and as a cue, setting a rhythm to the participant’s retrieval process. After the repetition phase, a serial recognition task was used to probe sentence memory. In this task, a sequence of two words appeared on the screen and the participant had to press either one of two buttons evaluating whether this sequence had appeared in the previous sentence.

As a further measure to ensure participants followed the instructions and actually engaged in inner retrieval during the thinking phase, the standard trial sequence was slightly modified for the 18 rehearsal check stimuli. These stimuli were presented pseudo-randomly during the experiment (one for each block). For each of these sentences, two of the fixation crosses in the thinking phase in the fourth and fifth position were substituted by the image of a microphone (cf. Figure 1). This probe was shown for 600 ms and cued the participant to repeat the respective word out fieldloud instead of silently, and their vocal response was recorded and stored in a sound file to be analyzed separately.

Each session was divided into nine blocks of eleven sentences each, with each block containing one attention check stimulus. After each block, the experiment was paused until the subject decided to resume. After the fifth block, participants were required to take a 5-min break. The duration of the whole experiment was approximately 35 min.

### Data Analysis

#### EEG Data

The EEG data were pre-processed with MATLAB (Version R2017a, Mathworks^®^, Natick, MA, United States) using “EEGLAB Toolbox” version 14 (Delorme and Makeig, 2004). The data from the two sessions of each subject were merged and each file was epochized in windows of 1600 ms (600 ms before stimulus onset and 1000 ms after) in order to capture local effects (short epochs). In order to capture the expected slow waves in the rehearsal phase, epochs of 7600 ms (600 ms before stimulus onset and 7000 ms after) were selected for all conditions (long epochs).

The data were manually cleaned to remove the most evident muscular artifacts and then re-referenced to the right mastoid. FASTER analysis (Nolan et al., 2010) was then run to remove blinks and eye-movement artifacts. This included high-pass filtering before the application of an ICA during the FASTER procedure. A 0.5 Hz high-pass filter (−6 dB cut-off frequencies of 0.25 Hz) as well as a notch filter at 50 Hz (bandwidth 3 Hz) to remove line noise, were applied for the short epochs, while a 0.03 Hz high-pass filter (−6 dB cut-off frequencies of 0.015 Hz), was applied for the long epochs. All epochs were low-pass filtered at 25 Hz (−6 dB cut-off frequencies of 21.875 Hz). The data were re-referenced to averaged mastoids and resampled to 1000 Hz.

For the statistical analysis, the “Fieldtrip toolbox” was used (Oostenveld et al., 2011). ERPs of the short epochs were calculated for each condition by averaging across subjects and by applying a baseline from 0 (onset of the stimulus) to 100 ms. We chose a post-stimulus baseline for all short epochs in order to have a uniform baseline across the reading and the rehearsal phase which would not be influenced by the rehearsal of the previous word. Baseline correction for the long epochs was applied between 500 ms before the onset to 0 ms. A non-parametric cluster-based permutation analysis was applied using dependent samples *t*-tests with the threshold for alpha fixed at 0.05. The minimum number of neighbourhood channels for a defined sample to be included in the statistic was equal to 2. A permutation test based on the Monte Carlo method (Maris and Oostenveld, 2007) was used with 1000 randomizations (α = 0.05). It should be noted that the cluster-based permutation test is reliable when it comes to identifying effects in the data, but does not allow for a precise identification of latency and distribution of these effects (Sassenhagen and Draschkow, 2019). Therefore, the time-windows and distributions reported in the result section are only those of the respective clusters identified via the test and do not necessarily reflect the exact time-windows and distributions of the effect.

#### Behavioral Data

Response accuracy in the decision task was calculated for each condition and then descriptive statistics were obtained using SPSS (IBM Corp. Released 2017. IBM SPSS Statistics for MacOS, Version 25.0. Armonk, NY, United States: IBM Corp.), and a Linear Mixed Effects (LME) analysis was carried out using the lme4 package (Bates et al., 2015) within R (R Core Team, 2018), with stimuli condition as a fixed effect and subject variability as a random effect. *P*-values were obtained by likelihood ratio tests comparing the full model against a null model. A series of *post hoc* pairwise *t*-test (Bonferroni corrected) were then completed. For the rehearsal check items, we evaluated the spoken responses of the participants and calculated the respective accuracy rates.

## Results

### Behavioral

**FIGURE 2:**
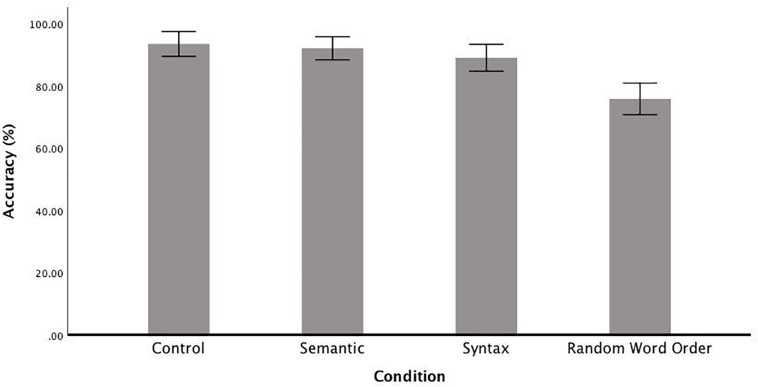
Mean accuracy rates of the serial recognition task by Condition (correct sentences (Control), random word order sentences (RWO), sentences with semantic anomalies (Semantic) and sentences with a syntactic gender agreement error (Syntax). Error bars show the 95% confidence interval.

Accuracy in the serial recognition task, in which participants had to decide whether a two-word sequence had been previously presented in the identical form, was above chance level in each of the conditions (Figure 2). The comparison between the full and the null models reveals that accuracy was affected by condition [χ^2^(1) = 77.03, *p* < 0.001]. To investigate this effect of condition, a series of *post hoc t*-test were carried out, which revealed no significant difference between the Control condition (*M* = 93%, *SD* = 9) and the Semantic condition (*M* = 92%, *SD* = 9) [*t*(23) = 1.11, *p* = 1.00], a significant difference between Control and Syntactic (*M* = 89%, *SD* = 10) conditions [*t*(23) = 3.12, *p* < 0.05] and a significant difference between the RWO condition (*M* = 76%, *SD* = 12) and all other conditions: Control [*t*(23) = 11.10, *p* < 0.001], Semantic [*t*(23) = 8.05, *p* < 0.001] and Syntactic [*t*(23) = 5.51, *p* < 0.001].

The rehearsal check items could unfortunately only be partly evaluated due to a technical error, due to which we recorded only responses up to 600 ms after production cue onset. Within that limited time window, participants produced an average of 51.3% (SD 15.5) correct and 8.2% (SD 5.0) incorrect answers.

### Event-Related Potentials

#### Reading Phase

**FIGURE 3:**
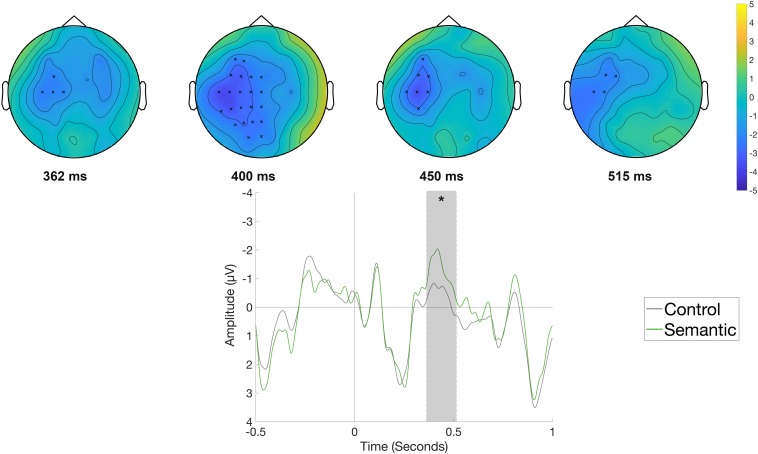
Significant clusters for the contrast Control versus Semantic in the reading phase at the object position. On the top topographic difference maps (Semantic – Control) of the effect are plotted across the time window (362 – 515 ms) indicated by the cluster based analysis; the electrodes belonging to the cluster are highlighted. On the bottom the waveform of the mean of the electrodes that are part of the cluster (F3, FC5, FC1, FC2, T7, C3, Cz, CP5, CP1, P3, Pz, F5, F1, FC3, FCz, C5, C1, CP3, CPz, P5, P1, PO3, TP7) – (^∗^*p* < 0.05) is plotted.

Figure 3 displays the waveforms and topographic difference maps elicited by the Semantic condition compared to the Control condition in the reading phase, time-locked to the onset of the object position (*e.g., Die Frau bindet den Schuh im Flur [The woman ties the shoe in the hallway.]*). The Semantic condition elicited a more negative-going waveform at left-central electrodes compared to the Control condition. Correspondingly, the cluster-based analysis showed a significant difference between the two conditions (*p* < 0.05), originating from a negative cluster observed at left-central sites beginning at around 360 ms and lasting until around 515 ms.

**FIGURE 4:**
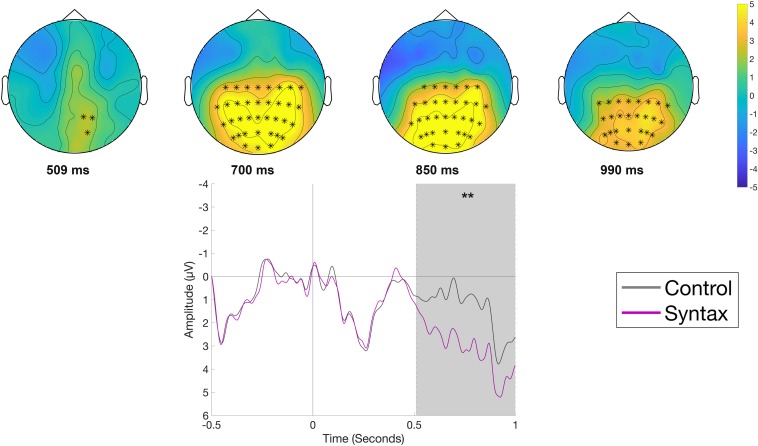
Significant clusters for the contrast Control versus Syntax in the reading phase at the object position. On the top topographic difference maps (Syntax – Control) of the effect are plotted across the time window (509 – 999 ms), indicated by the cluster based analysis; the electrodes belonging to the cluster are highlighted. On the bottom the waveform of the mean of the electrodes that are part of the cluster (C3, Cz, C4, CP5, CP1, CP2, CP6, P7, P3, Pz, P4, P8, POz, O1, Oz, O2, C1, C2, C6, CP3, CPz, CP4, P5, P1, P2, P6, PO5, PO3, PO4, PO6, TP8, PO7, PO8) – (^∗∗^*p* < 0.001) is plotted.

The comparison of the Syntactic and the Control condition (Figure 4), time-locked to the onset of the object position, indicated a more positive-going waveform at centro-posterior electrode sites for the Syntactic condition. The difference between conditions was significant (*p* < 0.01), with the effect corresponding to an observed positive cluster with a centro-posterior distribution and an approximate latency of 500–1000 ms. The observed timing and the distribution of the effects in the data, with a negativity for the semantic violation and a positivity for the syntactic violation, led us to categorize the observed ERP patterns as classical N400 and P600 components.

#### Rehearsal Phase

**FIGURE 5:**
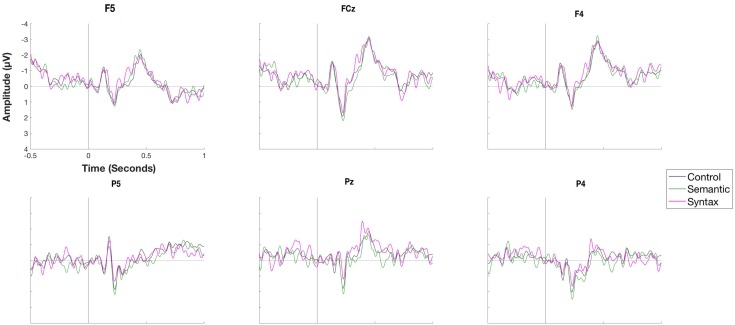
Waveforms of a selection of electrodes for the conditions Control, Semantic and Syntax for the object position during the rehearsal phase. No significant differences were observed.

**FIGURE 6:**
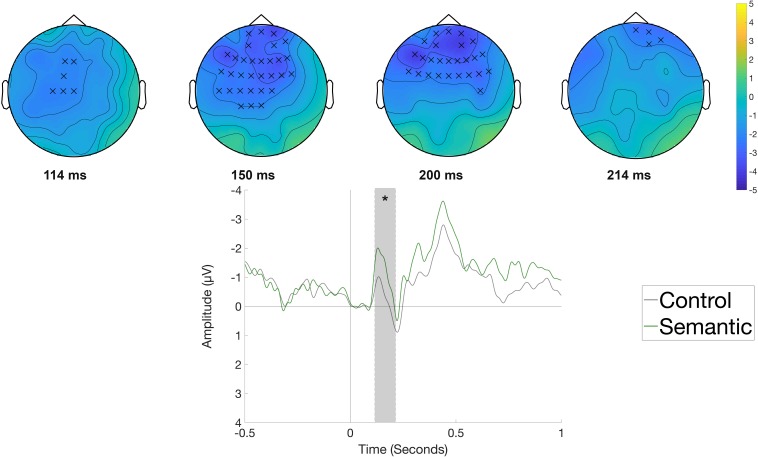
Significant clusters for the contrast Control versus Semantic in the rehearsal phase at the verb position. On the top topographic difference maps (Semantic – Control) of the effect are plotted across the time window (114 –214 ms) indicated by the cluster based analysis; the electrodes belonging to the cluster are highlighted. On the bottom the waveform of the mean of the electrodes that are part of the cluster (Fp1, Fpz, Fp2, F7, F3, Fz, F4, FC5, FC1, FC2, FC6, T7, C3, Cz, CP1, AF3, AF4, AF8, F5, F1, F2, F6, FC3, FCz, FC4, C5, C1, C2, CP3, CPz, FT7) – (^∗∗^*p* < 0.001) is plotted.

Figure 5 displays the waveforms elicited by the Semantic and Syntactic conditions in the rehearsal phase compared with the Control condition time-locked to the onset of the object (*e.g., Die Frau bindet den Schuh im Flur [The woman ties the shoe in the hallway.]*). The cluster-based permutation test did not reveal any significant differences between conditions. Figure 6 shows the waveforms and topographic difference maps of the contrast of the Control versus the Semantic condition at the verb position (*e.g.*, Die Frau bindet den Schuh im Flur *[The woman ties the shoe in the hallway.]*) in the rehearsal phase. The Semantic condition elicited a relatively early negative deflection compared to the Control condition at fronto-central electrode sites. The cluster-based analysis thus indicated a significant negative cluster (*p* < 0.05) between 114 and 214 ms with a fronto-central distribution.

**FIGURE 7:**
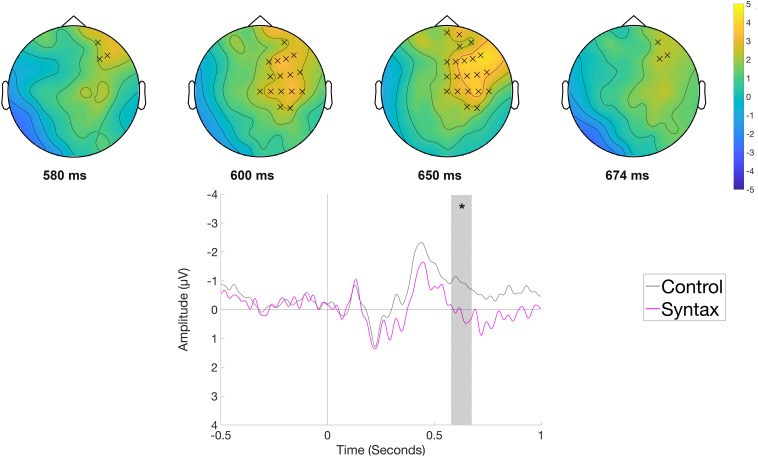
Significant clusters for the contrast Control versus Syntax in the rehearsal phase at the article position. On the top topographic difference maps (Syntax – Control) of the effect are plotted across the time window (580 – 674 ms) indicated by the cluster based analysis; the electrodes belonging to the cluster are highlighted. On the bottom the waveform of the mean of the electrodes that are part of the cluster (F4, F8, FC2, FC6, Cz, C4, T8, CP6, AF8, F2, F6, FC4, C2, C6, CP4, FT8) – (^∗^*p* < 0.05) is plotted.

The comparison of the Syntactic and the Control condition at the article position (*e.g.*, Die Frau bindet den Schuh im Flur *[The woman ties the shoe in the hallway.]*) indicated a right frontal positivity for syntactically anomalous sentences. When the entire epoch was taken into account, no significant clusters were found. The analysis of a narrower time window between 500 and 700 ms (selected *a priori* as a time window for the P600) showed a significant positive cluster (*p* < 0.05) between 580 and 674 ms with a right frontal distribution for the Syntactic condition (Figure 7). We would like to note that the significance of this effect depends on the application of high-pass filtering, which we chose in order to optimize ICA decomposition (cf. section EEG Data), while all other effects are also significant even when a more conservative filter (high-pass filter at 0.1 Hz, −6 dB, cut-off frequency 0.05 Hz) is applied.

#### Rehearsal Phase – Slow Waves

**FIGURE 8:**
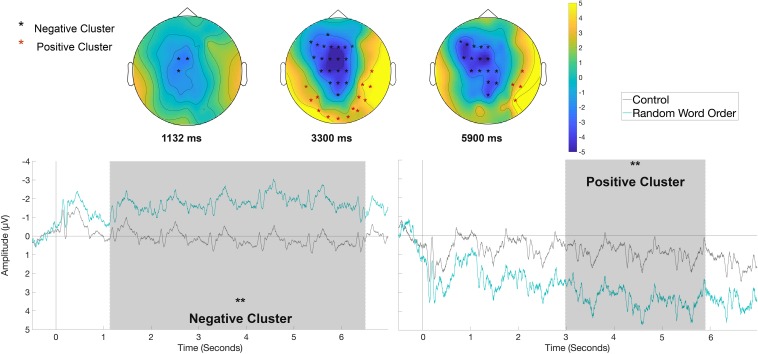
Significant clusters for the contrast Control versus Random Word Order in the rehearsal condition across the whole sentence. On the top topographic difference maps show the effects across the time windows of the negative cluster (1113 – 6500 ms) and of the positive cluster (2980 – 5900 ms) indicated by the cluster based analysis; the electrodes belonging to the cluster are highlighted. On the bottom the waveform of the mean of the electrodes that are part of the cluster (F7, F3, Fz, FC5, FC1, FC2, C3, Cz, CP1, CP2, Pz, AF3, F5, F1, F2, FC3, FCz, C1, C2, CPz) and of the positive cluster (T8, CP6, P7, P8, O1, Oz, O2, P5, P6, PO6, TP7, TP8, PO7, PO8) – (^∗∗^*p* < 0.01) are plotted.

For the analysis of the long epochs, given the nature of the slow waves, only clusters with significant time widows longer than 1 s (one-word time-window) will be reported. There were no significant clusters longer than 1 s for the contrasts between Control versus Semantic and Control versus Syntactic in the rehearsal phase. For the contrast Control versus Random Word Order, a significant negative cluster for the RWO condition(*p* < 0.01) was identified in the time window between 1130 and 6500 ms with fronto-central distribution and a positive cluster (*p* < 0.01) with right-posterior distribution in the time window between 2980 and 5900 ms, as displayed in Figure 8.

## Discussion

The present study aimed to specify the language production processes supporting subvocal rehearsal of sentences in a working memory task. In order to ensure that participants engaged in subvocal rehearsal, an overt articulation cue was presented intermittently, during which participants had to produce the respective words. Importantly, the cues appeared unpredictably in the middle of sentences making sure that participants did not know in advance which words would have to be spoken out loud and which ones, silently. In more than half of the overt articulation trials, participants repeated the correct words within the first 600 ms after the articulation cue, showing that they were largely following the task. Unfortunately, technical problems precluded the analysis of responses after 600 ms from the articulation cue. This is problematic as 600 ms is the typical onset latency in many articulation tasks (Indefrey and Levelt, 2004) and thus, we probably missed many potentially correct answers. Yet, even though the final performance in the overt production trials cannot be reported, we are confident that participants engaged in subvocal rehearsal as (i) the task instruction was to do so, (ii) the overt production trials ensured commitment to the task and (iii) subvocal rehearsal was a good strategy to be able to answer the questions that followed the rehearsal (presence of a given two-word sequence).

The performance in the serial recognition task replicates the sentence superiority effect and shows that sentences, independently of the presence of semantic or syntactic anomalies, are remembered better compared to ungrammatical word strings. Further, the ERP data show that rehearsal of unstructured word sequences compared to correct sentences was accompanied by a fronto-central negative shift covering 1.13 to 6.5 s and a bilateral posterior positivity between 2.98 and 5.9 s after rehearsal onset. In contrast, rehearsal of the semantic and syntactic violation conditions led to temporally and topographically different ERP responses at different sentence locations. In the semantic condition, a fronto-central negativity was found between 114 and 214 ms after the onset of the articulation cue at the position of the verb. In the syntactic condition, a positivity was found between 580 and 674 ms after the onset of the articulation cue for the syntactically incorrect determiner. In the following, we will first discuss the findings for each condition and then turn to outline the significance of the findings for conceptualizations of working memory and sentence production.

### Sentence Superiority Effect

Both behavioral and EEG data support increased processing costs for random strings of words. The accuracy in the serial recognition task was significantly better for sentences than for word sequences. ERPs were analyzed from the beginning of the repetition phase as the lack of structure and coherent meaning was assumed to induce an enhanced processing load from the start. Indeed, a long-lasting negative shift was evident from the onset of the cue for the second word until the onset of the cue for the last word. Additionally, a posteriorly distributed positive shift was observed, which started later and ended earlier compared to the negative shift. The gradual onset of the effects might be interpreted as an indication of the attentional demands building up gradually with the first two items probably still benefiting from a primacy effect (Ebbinghaus, 1913). The negativity consisted of a frontal and a parietal portion. Previous ERP studies on verbal working memory have reported similar slow potentials, which varied depending on cognitive load and the stimulus material used (Lang et al., 1992; Ruchkin et al., 1992, 1997, 1999; Murphy et al., 2006). Initially, the frontal negative slow wave has been related to subvocal rehearsal proper (Ruchkin et al., 1992). Later studies have shown that it is also found in conditions where rehearsal is blocked and thus, it has been suggested that it is rather related to higher order cognitive control processes involved in verbal working memory (Bosch et al., 2001; Murphy et al., 2006). In the present study, articulation was manipulated in a different way than in most studies. Instead of blocking subvocal rehearsal by articulatory suppression (Murphy et al., 2006), articulation was enforced in the rehearsal phase. As participants were instructed to rehearse (and controlled for task compliance) it can be assumed that rehearsal occurred equally across all conditions. This means that the negativities in our experiment cannot be explained by assuming subvocal rehearsal in the more difficult condition and no rehearsal for the easier correct sentences. This is in line with the previous studies that showed negative shifts that were sensitive to the stimulus material, but not dependent on the possibility of subvocal rehearsal. We suggest that the fronto-central shift represents the allocation of additional attentional resources in the light of the higher working memory demands. For example, it may reflect an upregulation of ‘multiple-demand’ cortical regions, that come on-line in response to increased task difficulty (Fedorenko et al., 2013; Geranmayeh et al., 2014; Sliwinska et al., 2017). One such region, the anterior insula, which is deeper but anatomically close to the inferior frontal gyrus, has been shown to be upregulated during sentence repetition tasks when comprehension of the sentence to be repeated is more difficult, due to degrading of the auditory signal, but not when the sentence is simple and easy to understand (Brownsett et al., 2014). The posterior slow waves, the negative and the positive shift might reflect more stimulus specific memory strategies. Posterior negative shifts have also been observed previously both for verbal and visual working memory tasks (Ruchkin et al., 1992; Bosch et al., 2001). Based on studies relating posterior slow potentials to visuo-spatial memory operations (e.g., Rösler et al., 1995a, b), Bosch et al. (2001) speculated that posterior slow potentials could be related to processes of transforming a visual to a phonological code. Bosch et al. (2001) also observed posterior positive shifts for visuo-spatial tasks in which no verbalization was possible. Thus, posterior slow waves could be related to image-based memory strategies. As in our experiment the words were presented visually, we tentatively adopt those ideas, namely that participants may both transform the visual code into a phonological one but that they also store the original visual code. Concerning the neural generation of slow waves, it has been suggested that signals from thalamic nuclei enhance the excitability of cortical areas, which Birbaumer et al. (1990) term “cerebral potentiality.” In this way, processing resources are allocated to specific cortical areas in preparation of a cognitive task (cf. Birbaumer et al., 1990). The interpretation of the slow waves in our study as reflecting the relatively enhanced attentional and strategic demands of the unstructured word lists is in concord with this model.

In sum, the sentence superiority effect is reflected in increased accuracy rates in a serial recognition task, and neurophysiologically, in a decrease in fronto-central and parietal slow waves which probably reflect enhanced costs in terms of cognitive control, visual and visual to phonological coding respectively.

### Lexical-Semantic Violations

While the memorization of unstructured word sequences seems to recruit domain-general networks that support working memory processing, as we argued above, the memorization of sentences that only include a semantic anomaly leads to different effects. Behaviorally, semantically anomalous sentences led to comparable accuracy rates in the serial recognition task as correct sentences. ERP analyses of the verb position revealed a significant early fronto-central negativity peaking around 150 ms after cue onset for verbs that are later followed by a semantically unexpected noun compared to verbs from normal sentences. At the noun position, which yielded a semantic violation N400 effect during reading, no significant ERP effects were found. We take the early position of the effect as well as its early latency as an indication of advance planning of the direct object at the stage of abstract lexical planning. An extensive review by Indefrey and Levelt (2004) estimated that lexical selection for single word production occurs from about 150 to 350 ms after onset of a production cue. In our sentence repetition task, we do not know exactly when lexical selection of the verb started, but the early ERP response that largely covers the assumed time frame of that process, suggests that the semantically anomalous word that comes right after created some kind of processing cost at the lexical selection stage of the verb. This implies that the lexical planning of the verb and its arguments occurs at the same time or in fast sequence. Previous studies suggest that lexical planning in sentence production occurs at a rather large scope, spanning at least a single phrase or more (Smith and Wheeldon, 1999; Martin et al., 2010; Klaus et al., 2017). The early negativity at the verb shows that the planning scope comprises at least two words in advance, or maybe the entire phrase. Its timing as well as its distribution are inconsistent with an interpretation as an N400, which occurs at a later time window and with a different distribution. Similar effects with similar early timings have recently been reported in studies using a picture-guided noun phrase elicitation paradigm (Pylkkänen et al., 2014; Blanco-Elorrieta et al., 2018). In these studies, participants composed adjective-noun combinations which were compared to the production of two single non-composable words. The two-word combinations led to significant effects as measured with MEG starting from ∼180 ms. The effects could be localized to ventro-medial frontal cortex and to antero-lateral temporal cortex and were related to semantic composition independent of spoken or signed modality (Pylkkänen et al., 2014; Blanco-Elorrieta et al., 2018). Similar effects of semantic composition were found during comprehension of equally simple noun phrases (Bemis and Pylkkanen, 2011; Neufeld et al., 2016). Compared to the negativities reported in these studies, our negativity seems to occur even earlier. Note that we used a sentence repetition task, implying that the single words were already retrieved a relatively short time before the production cue. The studies on language production cited above used a picture-guided elicitation task instead, whereby participants have to first interpret the picture correctly, then retrieve the respective word without the picture being shown (Pylkkänen et al., 2014; Blanco-Elorrieta et al., 2018). This task difference could lead to a shift in timing of the same or a similar effect. Note that no N400 was observed at the position of the actual semantic violation in our study. Yet, we know from the observed N400 in the reading phase that the direct object in the semantically anomalous condition induces semantic processing difficulties. This difference in the position and type of the ERP effect shows that the additional processing costs due to the semantically anomalous noun “have been paid before” during production and that there are no further integration difficulties at later stages.

### Syntactic Violations

Like with semantic anomalies, local ERP effects were found for the syntactically anomalous sentences during word-by-word silent production. Behaviorally, syntactically anomalous sentences led to slightly lower accuracy rates in the serial recognition task than correct sentences. During the reading phase, a P600 effect was found at the noun position, but this position did not yield a significant effect during the rehearsal phase. Here, an effect was found after cue onset for determiners that are incongruent with the subsequent noun, although only when the statistical analysis was restricted to a time window of interest. The effect was a positivity with a right-frontal distribution peaking around 600 ms. We take the sentential position of the effect as well as its late latency as an indication of a mismatch between the planned or already articulated determiner form and the determiner form required by the gender of the subsequent noun.

Models of language production assume that gender information is stored in the mental lexicon linked to the noun either at the level of abstract lexical forms (Dell, 1986; Levelt, 1989) or at the level of phonological word forms (Miozzo and Caramazza, 1997). This implies that the noun has to be accessed either in its abstract or phonological form before gender information can be accessed. A previous ERP study on the production of simple determiner-noun phrases provided evidence that the phonological form of the determiner is accessed during or even after the phonological planning of the noun (Bürki et al., 2016). For the interpretation of our effect, this means that the mismatch effect we observe at the determiner indicates that the noun has become activated at that position. Further, the late onset of the ERP is consistent with the possibility that the encoding level at which the effect occurs is phonological encoding or a later process. The idea is based on the sequential nature of word production processes with phonological encoding occurring at some point after 200 ms after onset of production planning (Indefrey and Levelt, 2004). Thus, it seems plausible that the processing difficulty at the determiner occurs at that stage or later. Note that this remains speculative because in principle, ERP components can be influenced by cognitive processes occurring some time before their onset, so the nature of the difficulty experienced at that level is difficult to determine on the basis of the observed ERP pattern: it could be an effort to override a syntactic rule and thus directly related to a linguistic process; or it could be due to domain-general processing difficulties that accompany the former process. A study of Jessen et al. (2017) observed a right frontal negative deflection in response to production of regular participle forms compared to irregular participle forms in German. Although our design is different in the sense that the production of a correct noun phrase is compared to the production of an incorrect one, both experiments share the comparison of a rule-guided condition to an exceptional condition. In both cases, the exceptional condition elicited a right-frontal positivity. Thus, the positivity for the syntactic violation in our case could reflect the costs for overriding the established rule that specifies the determiner form according to the noun’s gender. Another possibility would be that the effect is not related to determiner planning at all, but rather to production monitoring processes taking place in parallel. Shitova et al. (2017) reported a modulation of a relatively late occurring P300 component by complexity and task-switching during a noun phrase production task. They interpreted this effect as related to the allocation and use of processing resources in the face of the affordances of the production task. Our late positivity could be related to similar processes, namely the additional attentional costs that come about by producing an outright grammatical error while the grammatically correct form is simultaneously activated.

Even if the exact processing level reflected by the positivity for the gender incongruent determiner is difficult to determine, the fact that the effect occurs on the determiner shows that the processing difficulties induced by the syntactically incorrect form appear at a later planning stage than the problems induced by improper lexical-semantic choices.

### Implications for Models of Language Production and Working Memory

In the present study we used sentence repetition to tackle the problem of how different types of linguistic information assist working memory. By doing this we also tapped, at least partly, into sentence production. Earlier studies with adults and many studies with developmental populations have used sentence repetition to test language production processes (Rodd and Braine, 1971; Ferreira, 1991; Brownsett et al., 2014; Klem et al., 2015). Admittedly, sentence repetition does not correspond to natural sentence production as the full form and content are already clear from the beginning and thus, working memory may be taxed much more and access and selection processes somehow less. Yet, theoretic models of sentence repetition converge in the assumption that language processing plays an important role in this task. The conceptual regeneration hypothesis, for example, posits that for the most part conceptual representations of sentences are stored and that syntactic and phonological aspects are generated during the process of repetition (Potter and Lombardi, 1990; Lombardi and Potter, 1992). Similarly, it is assumed in the context of Baddeley’s multi-component model of working memory that the language processing system contributes to the advantage for memorizing sentences compared to word lists (Baddeley et al., 2009). Thus, sentence repetition may be a suitable method for assessing certain stages of language production. Obviously, due to the constrained nature of the task, the production process may not be entirely comparable to production in more natural contexts. The positions as well as the latencies of the ERP effects in response to silent rehearsal of our lexical-semantic and syntactic violations provide clear evidence that the respective types of information are accessed at different stages during the reproduction process. It is most plausible to assume that the respective violations created processing difficulties at those production levels where the critical information is in conflict with certain planning processes. The present findings are thus consistent with models of sentence production that assume different scopes of planning ahead for different types of information, as for example in the classic model of Garrett (1975, 1982), assuming at least a phrasal level of planning for abstract lexical forms and a smaller scope for concrete phonological realizations. By integrating this evidence with models of working memory, the findings support those models that explicitly include the language production architecture in their maintenance mechanisms (Cowan, 1999; Buchsbaum and D’Esposito, 2019). The costs for maintaining sentences are dramatically decreased compared to ungrammatical word sequences, even in the presence of some embedded semantic or syntactic violations. The random word sequences induced processing costs all the way, while the ungrammatical sentence conditions showed only indications of local processing costs. This is consistent with the idea that working memory makes use of an incremental multi-staged language production process that benefits from lexical-semantic and syntactic relations between sentence parts as they are continuously integrated.

## Conclusion

Using a silent cued repetition task, a methodology that to our knowledge has never been used at sentence level, we found a sentence superiority effect for recognizing word sequences in sentences, compared to unstructured word sequences. Sentences with local semantic or syntactic violations were remembered comparably well, close to correct sentences, with a minor disadvantage for syntactically incorrect sentences. Electrophysiologically, a fronto-central and posterior slow wave reflected enhanced processing costs for the unstructured linguistic strings. Semantically and syntactically anomalous sentences, in contrast, yielded rather local processing costs reflecting the respective sentence planning stages at which the difficulties occurred, most likely access of abstract lexical forms and later phonological or monitoring processes. The results can be best explained by assuming that subvocal rehearsal of sentences in working memory includes typical stages of sentence planning, in line with working memory models that integrate the language architecture as a powerful supporting system. Finally, since the reported ERP effects are novel and in the case of syntactic anomalies statistically fragile, a replication of the effects would be highly desirable. In principle, the paradigm could become a valuable add-on in the toolbox for the study of the neurophysiological basis of on-line sentence production.

## Data Availability Statement

The datasets generated for this study are available on request to the corresponding authors.

## Ethics Statement

The studies involving human participants were reviewed and approved by the Kommission für Forschungsethik (KFE) - University of Osnabrück. The patients/participants provided their written informed consent to participate in this study.

## Author Contributions

MM conducted the research, from an original idea of RZ, created the design of the study under the supervision of JM, RZ, and FV, and performed the data analysis under the supervision of JM, FV, and TG. JM and MM wrote the first draft of the manuscript. All authors contributed to the revision of the manuscript.

## Conflict of Interest

The authors declare that the research was conducted in the absence of any commercial or financial relationships that could be construed as a potential conflict of interest.
