# Supplementary material for: Distinct Neural Processes for Memorizing Form and Meaning Within Sentences
Source: Front Hum Neurosci. 2019 Dec 5;13:412. doi: 10.3389/fnhum.2019.00412 (PMC6906200; doi:10.3389/fnhum.2019.00412)
Supplement: Supplementary file 1 [file Data_Sheet_1.pdf]

Stimuli List

1 Die Katze isst den Vogel im Garten

2 Die Frau bindet den Schuh im Flur

3 Die Ente verspeist das Brot im See

4 Der Postbote leert den Briefkasten am Mittag

5 Die Friseurin öffnet den Salon am Nachmittag

6 Die Polizistin verhaftet den Dieb im Geschäft

7 Der Maler verkauft das Bild ziemlich teuer

8 Der Schreiner haut den Nagel wirklich kräftig

9 Der Rentner lobt den Enkel immer wieder

10 Ein Affe kratzt den Rücken des Weibchens

11 Der Schiedsrichter sieht das Foul im Fußballfeld

12 Der Schauspieler liest das Drehbuch im Ankleideraum

13 Der Koch bringt das Essen sehr zügig

14 Der Pilot steuert das Flugzeug im Gewitter

15 Ein Arbeiter wechselt den Motor eines Autos

16 Ein Adler erfasst den Spatz im Sturzflug

17 Eine Sportlerin überspringt das Hindernis ohne Verletzung

18 Ein Zuschauer verschläft den Film im Kino

19 Ein Dinosaurier trinkt das Wasser des Teiches

20 Der Angestellte zählt das Geld des Kunden

21 Der Hund beißt den Besitzer wirklich unerwartet

22 Der Gast verbummelt den Schlüssel am Empfangsbüro

23 Die Maurer bauen das Haus im Dorf

24 Der Zugführer kontrolliert das Ticket des Mannes

25 Das Boot verlässt den Hafen jeden Abend

26 Ein Kaninchen futtert den Salat im Wald

27 Die Chefin entlässtden Mitarbeiter ohne Reue

28 Der Arbeitnehmer druckt das Dokument zum Unterzeichnen

29 Der Bauer schlachtet das Huhn im Hof

30 Das Mädchen rennt den Weg zum Spielplatz

31 Eine Försterin markiert den Baum in rot

32 Ein Räuberbedroht den Kassierer im Kiosk

33 Ein Bettler holt das Brot im Laden

34 Das Chamäleon verschluckt das Insekt im Gras

35 Der Kurier liefert das Paket zum Haus

36 Der Wanderer entzündet das Feuer am Wegrand

37 Eine Laterne beleuchtet den Parkplatz des Marktes

38 Der Bodyguard schützt den Prominenten im Theater

39 Die Journalistin schreibt den Artikel am Smartphone

40 Der Jäger schießt den Hasen mit Schrot

41 Der Täter erwürgt das Opfer von hinten

42 Der Priester erhält den Brief vom Bischof

43 Der Redakteur korrigiert den Text des Journalisten

44 Die Gazelle gebärt das Junge im Zoo

45 Der Wirt verschüttet das Bier am Tresen

46 Die Freundin kauft das Geschenk zum Geburtstag

47 Der Detektiv schnappt den Mörderder Paare

48 Der Autor präsentiert das Manuskript im Fernsehen

49 Der Clown grüßt das Publikum des Zirkus

50 Ein Seemann reinigt das Schiff im Hafen

51 Die Leoparden jagen das Zebra zu zweit

52 Ein Angler fängt den Fisch zum Abendessen

53 Der Junge wirft das Geldstück weit fort

54 Der Arzt unterschreibt das Rezept gegen Allergien

55 Die Lehrerin ermahnt den Schüler sehr häufig

56 Der Hersteller stellt das Produkt ins Internet

57 Die Trainerin hebt das Gewicht wirklich mühelos

58 Die Mutter erzählt das Märchen immer wieder

59 Der Filmregisseur gewinnt den Preis in Berlin

60 Der Schneider näht den Rock der Diva

61 Der Hausmeister repariert das Fenster des Wohnheims

62 Das Kind tritt den Ball viele Male

63 Der Klemptner sägt das Rohr der Spüle

64 Der Verkäufer schließt den Laden jeden Abend

65 Der Anwalt überzeugt den Richter mit Argumenten

66 Das Pferd bekommt den Zucker vom Pfleger

67 Der Metzger kocht das Fleisch des Schweins

68 Der Onkel besucht den Neffen jedes Jahr

69 Ein Schwimmer kreuzt das Becken des Freibads

70 Der Butler begleitet den Köning überall hin

71 Die Künstlerin zeigt das Foto ihrer Galerie

72 Die Professorin erwartet den Studenten im Büro

73 Der Hirte scheucht das Schaf ganz schleunig

74 Der Designer entwirft das Logo mit Photoshop

75 Der Assistent unterstützt den Professor im Unterricht

76 Ein Besucher betrachtet das Kunstwerk im Museum

77 Die Kunden verhandeln den Preis der Fernseher

78 Ein Inspektor betritt das Restaurant zur Sanitärprüfung

79 Der Psycholinguist plant das Experiment im Labor

80 Die Kamele verlassen den Zoo heute Abend

81 Der Betrunkene zerbricht das Glas ohne Absicht

82 Der Soldat besiegt den Angreifer am Boden

83 Der Ritter gibt das Schwert mit Vorsicht

84 Der Tiger attackiert den Trainer im Zirkus

85 Der Junge schluckt den Kaugummi aus Versehen

86 Der Hofnarr jongliert den Ball am Marktplatz

87 Ein Müller verarbeitet das Korn zu Mehl

88 Der Spieler erringt den Sieg des Turniers

89 Der Vogel singt das Lied im Busch

90 Der Musiker spielt das Cello im Schloss

101 Die Katze zerbricht den Vogel im Garten

102 Die Frau steuert den Schuh im Flur

103 Die Ente besucht das Brot im See

104 Der Postbote sieht den Briefkasten am Mittag

105 Die Friseurin näht den Salon am Nachmittag

106 Die Polizistin entzündet den Dieb im Geschäft

107 Der Maler verschüttet das Bild ziemlich teuer

108 Der Schreiner schützt den Nagel wirklich kräftig

109 Der Rentner erringt den Enkel immer wieder

110 Ein Affe holt den Rücken des Weibchens

111 Der Schiedsrichter kreuzt das Foul im Fußballfeld

112 Der Schauspieler verhaftet das Drehbuch im Ankleideraum

113 Der Koch liest das Essen sehr zügig

114 Der Pilot fängt das Flugzeug im Gewitter

115 Ein Arbeiter unterschreibt den Motor eines Autos

116 Ein Adler gibt den Spatz im Sturzflug

117 Eine Sportlerin sägt das Hindernis ohne Verletzung

118 Ein Zuschauer stellt den Film im Kino

119 Ein Dinosaurier besiegt das Wasser des Teiches

120 Der Angestellte singt das Geld des Kunden

121 Der Hund spielt den Besitzer wirklich unerwartet

122 Der Gast erwürgt den Schlüssel am Empfangsbüro

123 Die Maurer beißt das Haus im Dorf

124 Der Zugführer verlässt das Ticket des Mannes

125 Das Boot bekommt den Hafen jeden Abend

126 Ein Kaninchen baut den Salat im Wald

127 Die Chefin entwirft den Mitarbeiter ohne Reue

128 Der Arbeitnehmer tritt das Dokument zum Unterzeichnen

129 Der Bauer plant das Huhn im Hof

130 Das Mädchen bringt den Weg zum Spielplatz

131 Eine Försterin jagt den Baum in Rot

132 Ein Räuber repariert den Kassierer im Kiosk

133 Ein Bettler verschläft das Brot im Laden

134 Das Chamäleon wechselt das Insekt im Gras

135 Der Kurier korrigiert das Paket zum Haus

136 Der Wanderer haut das Feuer am Wegrand

137 Eine Laterne wirft den Parkplatz des Marktes

138 Der Bodyguard kauft den Prominenten im Theater

139 Die Journalistin hebt den Artikel am Smartphone

140 Der Jäger rennt den Hasen mit Schrot

141 Der Täter verkauft das Opfer von hinten

142 Der Priester reinigt den Brief vom Bischof

143 Der Redakteur kocht den Text des Journalisten

144 Die Gazelle schluckt das Junge im Zoo

145 Der Wirt entlässtdas Bier am Tresen

146 Die Freundin kratzt das Geschenk zum Geburtstag

147 Der Detektiv markiert den Mörderder Paare

148 Der Autor bedroht das Manuskript im Fernsehen

149 Der Clown jongliert das Publikum des Zirkus

150 Ein Seemann schreibt das Schiff im Hafen

151 Die Leoparden trinken das Zebra zu zweit

152 Ein Angler betritt den Fisch zum Abendessen

153 Der Junge erwartet das Geldstück weit fort

154 Der Arzt gewinnt das Rezept gegen Allergien

155 Die Lehrerin verhandelt den Schüler sehr häufig

156 Der Hersteller grüßt das Produkt ins Internet

157 Die Trainerin betrachtet das Gewicht wirklich mühelos

158 Die Mutter kontrolliert das Märchen immer wieder

159 Der Filmregisseur zeigt den Preis in Berlin

160 Der Schneider begleitet den Rock der Diva

161 Der Hausmeister isst das Fensterdes Wohnheims

162 Das Kind unterstützt den Ball viele Male

163 Der Klempner ermahnt das Rohr der Spüle

164 Der Verkäufer druckt den Laden jeden Abend

165 Der Anwaltleert den Richtermit Argumenten

166 Das Pferd scheucht den Zucker vom Pfleger

167 Der Metzger erzählt das Fleischdes Schweins

168 Der Onkel beleuchtet den Neffen jedes Jahr

169 Ein Schwimmer erfasst das Beckendes Freibads

170 Der Butler verbummelt den König überall hin

171 Die Künstlerin schnappt das Foto ihrer Galerie

172 Die Professorin erhält den Studenten im Büro

173 Der Hirte verschluckt das Schaf ganz schleunig

174 Der Designer zählt das Logo mit Photoshop

175 Der Assistent liefert den Professor im Unterricht

176 Ein Besucher schießt das Kunstwerk im Museum

177 Die Kunden verlassen den Preis der Fernseher

178 Ein Inspektor verarbeitet das Restaurant zur Sanitärprüfung

179 Der Psycholinguist überzeugt das Experiment im Labor

180 Die Kamele futtern den Zoo heute Abend

181 Der Betrunkene schlachtet das Glas ohne Absicht

182 Der Soldat lobt den Angreifer am Boden

183 Der Ritter öffnet das Schwert mit Vorsicht

184 Der Tiger gebärt den Trainer im Zirkus

185 Der Junge präsentiert den Kaugummi aus Versehen

186 Der Hofnarr schließt den Ball am Marktplatz

187 Der Müller überspringt das Korn zu Mehl

188 Der Spieler bindet den Sieg des Turniers

189 Der Vogel verspeist das Lied im Busch

190 Der Musiker attackiert das Cello im Schloss

201 Die Katze isst das Vogel im Garten

202 Die Frau bindet das Schuh im Flur

203 Die Ente verspeist den Brot im See

204 Der Postbote leert das Briefkasten am Mittag

205 Die Friseurin öffnet das Salon am Nachmittag

206 Die Polizistin verhaftet das Dieb im Geschäft

207 Der Maler verkauft den Bild ziemlich teuer

208 Der Schreiner haut das Nagel wirklich kräftig

209 Der Rentner lobt das Enkel immer wieder

210 Ein Affe kratzt das Rücken des Weibchens

211 Der Schiedsrichter sieht den Foul im Fußballfeld

212 Der Schauspieler liest den Drehbuch im Ankleideraum

213 Der Koch befördert den Essen sehr zügig/scharf

214 Der Pilot steuert den Flugzeug im Gewitter

215 Ein Arbeiter wechselt das Motor eines Autos

216 Ein Adler erfasst das Spatz im Sturzflug

217 Eine Sportlerin überspringt den Hindernis ohne Verletzung

218 Ein Zuschauer verschläft das Film im Kino

219 Ein Dinosaurier trinkt den Wasser des Teiches

220 Der Angestellte zählt den Geld des Kunden

221 Der Hund beißt das Besitzer wirklich unerwartet

222 Der Gast verbummelt das Schlüssel am Empfangsbüro

223 Die Maurer bauen den Haus im Dorf

224 Der Zugführer kontrolliert den Ticket des Mannes

225 Das Boot verlässt das Hafen jeden Abend

226 Ein Kaninchen futtert das Salat im Wald

227 Die Chefin entlässt das Mitarbeiter ohne Reue

228 Der Arbeitnehmer druckt den Dokument zum Unterzeichnen

229 Der Bauer schlachtet den Huhn im Hof

230 Das Mädchen rennt das Weg zum Spielplatz

231 Eine Försterin markiert das Baum in Rot

232 Ein Räuber bedroht das Kassierer im Kiosk

233 Ein Bettler holt den Brot im Laden

234 Das Chamäleon verschluckt den Insekt im Gras

235 Der Kurier liefert den Paket zum Haus

236 Der Wanderer entzündet den Feuer am Wegrand

237 Eine Lanterne beleuchtet das Parkplatz des Marktes

238 Der Bodyguard schützt das Prominenten im Geschäft

239 Die Journalistin schreibt das Artikel am Smartphone

240 Der Jäger schießt den Schwein mit Schrot

241 Der Täter erwürgt den Opfer von hinten

242 Der Priester erhält das Brief vom Bischof

243 Der Redakteur korrigiert das Text des Journalisten

244 Die Gazelle gebärt den Junge im Zoo

245 Der Wirt verschüttet den Bier am Tresen

246 Die Freundin kauft den Geschenk zum Geburstag

247 Der Detektiv schnappt das Mörderder Paare

248 Der Autor präsentiert den Manuskript im Fernsehen

249 Der Clown grüßt den Publikum des Zirkus

250 Ein Seemann reinigt den Schiff im Hafen

251 Die Leoparden jagen den Zebra zu zweit

252 Ein Angler fängt das Fisch zur Abendessen

253 Der Junge wirft den Geldstück weit fort

254 Der Arzt unterschreibt den Rezept gegen Allergien

255 Die Lehrerin ermahnt das Schüler sehr häufig

256 Der Hersteller stellt den Produkt ins Internet

257 Die Trainerin hebt den Gewicht wirklich mühelos

258 Die Mutter erzählt den Märchen immer wieder

259 Der Filmregisseur gewinnt das Preis in Berlin

260 Der Schneider näht das Rock der Diva

261 Der Hausmeister repariert den Fenster des Wohnheims

262 Das Kind tritt das Ball viele Male

263 Der Klempner sägt den Rohr der Spüle

264 Der Verkäufer schließt das Laden jeden Abend

265 Der Anwalt überzeugt das Richter mit Argumenten

266 Das Pferd bekommt das Zucker vom Pfleger

267 Der Metzger kocht den Fleisch des Schweins

268 Der Onkel besucht das Neffen jedes Jahr

269 Ein Schwimmer kreuzt den Becken des Freibads

270 Der Butler begleitet das König überall hin

271 Die Künstlerin zeigt den Foto ihrer Galerie

272 Die Professorin erwartet das Studenten im Büro

273 Der Hirte scheucht den Schaf ganz schleunig

274 Der Designer entwirft den Logo mit Photoshop

275 Der Assistent unterstützt das Professor im Unterricht

276 Ein Besucher betrachtet den Kunstwerk im Museum

277 Die Kunden verhandeln den Angebot der Fernseher

278 Ein Inspektor betritt den Restaurant zur Sanitärprüfung

279 Der Psycholinguist plant den Experiment im Labor

280 Die Kamele verlassen das Zoo heute Abend

281 Der Betrunkene zerbricht den Glas ohne Absicht

282 Der Soldat besiegt das Angreifer am Boden

283 Der Ritter gibt den Schwert mit Vorsicht

284 Der Tiger attackiert das Trainer im Zirkus

285 Der Junge schluckt den Kaugummi aus Versehen

286 Der Hofnarr jongliert das Ball sehr geschickt

287 Der Müller verarbeitet den Korn zu Mehl

288 Der Spieler erringt das Sieg des Turniers

289 Der Vogel singt den Lied im Busch

290 Der Musiker spielt den Cello im Schloss

301 Die im Vogel Garten isst Katze den

302 Frau den Die Flur bindet Schuh im

303 See im Brot Ente das verspeist Die

304 Postbote Mittag den leert am Briefkasten Der

305 am den Salon Nachmittag öffnet Friseurin Die

306 Die Dieb im verhaftet Polizistin Geschäft den

307 Maler teuer Der das verkauft ziemlich Bild

308 Schreiner den Der kräftig haut wirklich Nagel

309 wieder Rentner lobt immer Enkel den Der

310 Weibchens Ein des Affe Rücken kratzt den

311 Der im das sieht Foul Fußballfeld Schiedsrichter

312 Der das im liest Ankleideraum Schauspieler Drehbuch

313 Der das sehr bringt zügig Koch Essen

314 Der das im steuert Gewitter Pilot Flugzeug

315 wechselt eines Autos Ein Arbeiter Motor den

316 erfasst im Sturzflug Ein Adler Spatz den

317 überspringt ohne Verletzung Eine Sportlerin Hindernis das

318 Kino im Zuschauer Ein verschläft Film den

319 Teiches des Dinosaurier Ein trinkt Wasser das

320 Kunden des Angestellte Der zählt Geld das

321 beißt unerwartet Besitzer wirklich Der den Hund

322 verbummelt Empfangsbüro Schlüssel am Der den Gast

323 bauen Dorf Haus im Die das Maurer

324 das Der Mannes des kontrolliert Zugführer Ticket

325 den Das Abend jeden verlässt Boot Hafen

326 den Ein Wald im futtert Kaninchen Salat

327 den ohne entlässt Reue Mitarbeiter Chefin Die

328 das zum druckt Unterzeichnen Dokument Arbeitnehmer Der

329 das im schlachtet Hof Huhn Bauer Der

330 Spielplatz zum Das den rennt Mädchen Weg

331 rot in Eine den markiert Försterin Baum

332 Kiosk im Ein den bedroht Räuber Kassierer

333 Bettler Brot das im holt Ein Laden

334 Chamäleon Insekt das im verschluckt Das Gras

335 Kurier Paket das zum liefert Der Haus

336 Der das am entzündet Wegrand Feuer Wanderer

337 Eine den des beleuchtet Marktes Parkplatz Laterne

338 Der den im schützt Theater Prominenten Bodyguard

339 Smartphone den schreibt am Die Artikel Journalistin

340 Schrot den schießt mit Der Hasen Jäger

341 hinten das erwürgt von Der Opfer Täter

342 Der vom Brief Bischof erhält Priester den

343 Der des Text Journalisten korrigiert Redakteur den

344 Die im Junge Zoo gebärt Gazelle das

345 Der das Tresen verschüttet am Wirt Bier

346 Die das Geburstag kauft zum Freundin Geschenk

347 Der den Paare schnappt der Detektiv Mörder

348 Autor Der Fernsehen im das präsentiert Manuskript

349 Clown Der Zirkus des das grüßt Publikum

350 Seemann Ein Hafen im das reinigt Schiff

351 zu Die jagen zweit Leoparden Zebra das

352 zum Ein fängt Abendessen Angler Fisch den

353 weit Der wirft fort Junge Geldstück das

354 das gegen unterschreibt Allergien Der Rezept Arzt

355 den sehr ermahnt häufig Die Schüler Lehrerin

356 das ins stellt Internet Der Produkt Hersteller

357 Die das mühelos wirklich hebt Trainerin Gewicht

358 Die das wieder immer erzählt Mutter Märchen

359 Der den Berlin in gewinnt Filmregisseur Preis

360 Der Rock Diva den der näht Schneider

361 Der Fenster Wohnheims das des repariert Hausmeister

362 Das Ball Male den viele tritt Kind

363 das Rohr Spüle der sägt Klemptner Der

364 den Laden Abend jeden schließt Verkäufer Der

365 den Richter Argumenten mit überzeugt Anwalt Der

366 Pferd den vom bekommt Pfleger Zucker Das

367 Metzger das des kocht Schweins Fleisch Der

368 Onkel den jedes besucht Jahr Neffen Der

369 das Ein kreuzt Becken des Freibads Schwimmer

370 den Der begleitet Köning überall hin Butler

371 das Die zeigt Foto ihrer Galerie Künstlerin

372 Professorin Die erwartet Studenten Büro den im

373 Hirte Der scheucht Schaf schleunig das ganz

374 Designer Der entwirft Logo Photoshop das mit

375 den Der im unterstützt Unterricht Professor Assistent

376 das Ein im betrachtet Museum Kunstwerk Besucher

377 den Die der verhandeln Fernseher Preis Kunden

378 Ein Restaurant Sanitärprüfung zur das betritt Inspektor

379 Der Experiment Labor im das plant Psycholinguist

380 Die Zoo Abend heute den verlassen Kamele

381 Glas Der ohne zerbricht Absicht Betrunkene das

382 Angreifer Der am besiegt Boden Soldat den

383 Schwert Der mit gibt Vorsicht Ritter das

384 Zirkus Der den im attackiert Tiger Trainer

385 Versehen Der den aus schluckt Junge Kaugummi

386 Marktplatz Der den am jongliert Hofnarr Ball

387 Mehl Ein das zu verarbeitet Müller Korn

388 Der Sieg den des erringt Turniers Spieler

389 Der Lied das im singt Busch Vogel

390 Der Cello das im spielt Schloss Musiker

501 Der Stürmer schießt den Ball ins Tor

502 Die Studentin hält das Referat im Seminarraum

503 Der Verteidiger foult den Angreifer im Strafraum

504 Das Model läuft den Laufsteg elegant hinunter

505 Der Basketball trifft den Korb am Rand

506 Der Affe verspeist das Obst im Baum

507 Der Tischler sägt das Holz in Stücke

508 Die Diebin klaut das Fahrrad im Keller

509 Der Sprecher verkündet das Neueste vom Tag

510 Das Baby erblickt den Licht der Welt

511 Der Kaiser regiert den Volk mit Gnade

512 Der Leutnant kommandiert den Heer im Krieg

513 Der Haushälter kocht den Müll nach draußen

514 Die Frau besucht den Mantel im Internet

515 Eine Pflegerin bügelt den Senioren am Morgen

516 Lehrerin Die den beantwortet der Brief Eltern

517 schneidet Haar Der Salon im Friseur das

518	Ein	das	im	Schlagzeug	spielt	Musiker	Studio

Legend:

1-90 **Control stimuli**

101-190 **Semantically violated stimuli**

201-290 **Syntactically violated stimuli**

301-390 **Random Word Order stimuli**

501-518 **Rehearsal Check stimuli**
